# Bone marrow-derived mesenchymal stem cells induced by inflammatory cytokines produce angiogenetic factors and promote prostate cancer growth

**DOI:** 10.1186/s12885-017-3879-z

**Published:** 2017-12-21

**Authors:** Ke-Qin Yang, Yan Liu, Qing-Hua Huang, Ning Mo, Qing-Yun Zhang, Qing-Gui Meng, Ji-Wen Cheng

**Affiliations:** 1 Department of Urology, Affiliated Tumor Hospital of Guangxi Medical University, Nanning, Guangxi Zhuang Autonomous Region 530021 China; 2 Department of Orthopedics, Guiping People’s Hospital, Guiping, Guangxi Zhuang Autonomous Region 537200 China; 3 The Fifth Department of Chemotherapy, Affiliated Tumor Hospital of Guangxi Medical University, Nanning, Guangxi Zhuang Autonomous Region 530021 China; 4 Department of Breast Surgery, Affiliated Tumor Hospital of Guangxi Medical University, Nanning, Guangxi Zhuang Autonomous Region 530021 China; 5 Department of Urology, The First Affiliated Hospital of Guangxi Medical University, Nanning, Guangxi Zhuang Autonomous Region 530021 China

**Keywords:** Prostate cancer, Mesenchymal stem cells, Angiogenesis, HIF-1α, NRF2, Inflammation, PDGF, VEGF

## Abstract

**Background:**

Prostate is susceptible to infection and pro-inflammatory agents in a man’s whole life. Chronic inflammation might play important roles in the development and progression of prostate cancer. Mesenchymal stem cells (MSCs) are often recruited to the tumor microenvironment due to local inflammation. We have asked whether stimulation of MSCs by pro-inflammatory cytokines could promote prostate tumor growth. The current study investigated the possible involvement of MSCs stimulated by pro-inflammatory cytokines in promotion and angiogenesis of prostate cancer through relative pathway in vitro and in vivo.

**Methods:**

A syngeneic mouse model of C57 was established. The murine prostate cancer cells (RM-1) mixing with MSCs treated with tumor necrosis factor alpha (TNF-α) and interferon gamma (IFN-γ) or vehicle were subcutaneously injected into C57 mice. Tumor volume of C57 mouse model was estimated and serum level of platelet-derived growth factor (PDGF) and vascular endothelial growth factor (VEGF) was test by Enzyme-linked Immunosorbent Assay (ELISA). A hen egg test-chorioallantoic membrane (HET-CAM) assay was applied to test the effect of conditioned media of stimulated MSCs in chorioallantoic membrane angiogenesis. Short interfering RNA (siRNA) knocked down either hypoxia-inducible factor-1alpha (HIF-1α) or nuclear factor-erythroid-2-related factor 2 (NRF2) were employed. mRNA of PDGF and VEGF in MSCs, as well as NRF2 and HIF-1α was test by Real time polymerase chain reaction (PCR) analyses. Protein expression levels of PDGF and VEGF from conditioned medium, NRF2, HIF-1α, as well as PDGF and VEGF in MSCs were detected by Western blot analysis.

**Results:**

MSCs treated with TNF-α and IFN-γ promote tumor growth in C57 syngeneic mouse model, correlating with increased serum level of PDGF, VEGF. HET-CAM assay shows the angiogenic effect of conditioned medium of MSCs pre-treated with the pro-inflammatory cytokines. mRNA and protein levels of two pro-angiogenic factors (PDGF and VEGF) and key hypoxia regulators (HIF-1α and NRF2) in MSCs were induced after MSCs’ pretreatment. siRNA knockdown either HIF-1α or NRF2 results reduction of PDGF and VEGF expression.

**Conclusions:**

MSCs stimulated by pro-inflammatory cytokines increase the expression of PDGF and VEGF via the NRF2-HIF-1α pathway and accelerate prostate cancer growth in mice.

**Electronic supplementary material:**

The online version of this article (10.1186/s12885-017-3879-z) contains supplementary material, which is available to authorized users.

## Background

Prostate cancer (PCa) is the first most frequently diagnosed type of cancer and the second most common cause of cancer death in men in the United States [1]. Growing evidence suggests that chronic inflammation might play important roles in the development and progression of PCa [2]. Consistently, lymphocytes infiltration and raised production of pro-inflammatory cytokines are commonly found in prostate cancer [3, 4].

Solid tumors are believed to be composed of tumor cells and non-tumorous, supportive cells that are commonly termed “tumor stroma”. Tumor stroma include cancer-associated fibroblasts, bone marrow-derived mesenchymal stem cells (BM-MSCs), smooth muscle cells, and various inflammatory cells such as lymphocytes, endothelial cells, macrophages [5]. These cells, as a whole, are known as tumor microenvironment, which has profound impact on cancer progression. Stromal cells can influence tumor growth and invasion through direct contact or the production of cytokines, growth factors and chemokines [6–8]. For example, several pro-inflammatory cytokines, including interferon gamma (IFN-γ), tumor necrosis factor alpha (TNF-α), transforming growth factor beta (TGF-β), and interleukin-10 (IL-10), have been shown to contribute to both the initiation and development of cancer [9–11].

Lately, the status of one class of stromal cells, the mesenchymal stem cells (MSCs), during cancer progression is emerging. MSCs, also known as multipotent adult stem cells of mesodermal germ layer origin, are multipotent stromal precursors that possess an innate ability for self-renewal and differentiation into cells of the osteogenic, adipogenic, and chondrogenic lineages. MSCs are often found in tumors of an inflammatory microenvironment, such as those in prostatic lesions. Studies have shown that MSCs modulate many aspects of tumorigenesis including tumor proliferation, angiogenesis, migration and metastasis and generate an immunosuppressive microenvironment [12]. Among them, angiogenesis of tumor is a major factor in tumor growth and progression.

Hypoxia is a common phenomenon at the site of tissue inflammatory [13]. Hypoxia is also considered a crucial promoting factor for angiogenesis through induction of hypoxia-inducible factor-1alpha (HIF-1α). Therefore, we hypothesis HIF-1α may be implicated in inflammation, whose overexpression can activates vascular endothelial growth factor (VEGF) and platelet-derived growth factor (PDGF) that facilitate the tumor angiogenesis. Nuclear factor-erythroid-2-related factor 2 (NRF2) is a upstream regulator of HIF-1α and plays a critical role in the cellular defense against oxidative stress [14]. Emerging data has revealed that NRF2 might not only protect normal cells from transforming into cancer cells, but also promote the cancer cells’ survival. NRF2 expression was shown to correlate with the tumorigenesis and progression of many tumors, including hepatocellular carcinoma [15], esophageal squamous cancer [16], colon tumor [17], and advanced lung cancer [18]. However, the precise role of NRF2 in inflammation and tumor angiogenesis remains unclear.

Considering the inherent tropism of MSCs for tumor tissue based on the inflammatory microenvironment and the pivotal role of chronic inflammation in the initiating and promoting of PCa [19, 20], we hypothesized that MSCs might play a significant role in development of PCa in the tumor microenvironment, possibly through promoting the formation of tumor vasculature.

In current study, we show that MSCs stimulated with the pro-inflammatory cytokines promote prostate tumor growth in mice and vasculature formation in chicken embryos. We further show that treatment of pro-inflammatory cytokines results in increased mRNA and protein levels of two key hypoxia regulators (HIF-1α and NRF2) in MSCs, and in increased mRNA and supernatant protein level of pro-angiogenic factors (PDGF and VEGF) of MSCs as well. The induction of PDGF and VEGF is not observed when the expression of HIF-1α and NRF2 is abolished. These results suggest a model that MSCs in an inflammatory microenvironment promote prostate cancer growth through increased angiogenesis by producing PDGF and VEGF in an NRF2-HIF-1α-dependent manner.

## Methods

### Cells and animals

RM-1, a murine prostate cancer cell line, was obtained from Tumor Immunology and Gene Therapy Center, Eastern Hepatobiliary Surgery Hospital (Shanghai, China). The cells were cultured in RPMI-1640 (BI) with 10% fetal bovine serum (FBS), supplemented with 2 mM L-glutamine and antibiotics (100 mg/ml of streptomycin and 100 units/ml of penicillin, all from Invitrogen), and in a humidified atmosphere at 37 °C with 5% of CO_2_. Cells were re-expended every 3 days in 70-80% confluence.

Male C57 mice (4~8-week old) were purchased from the Shanghai Experimental Animal Center of the Chinese Academy of Sciences (Shanghai, China). C57 mice were housed in pathogen-free conditions, and used to obtain MSCs. PCa model was established by using C57 mice as well. All procedures were performed upon the guidelines of the Committee on Animals of the Chinese Academy of Sciences. All animal studies were approved by the Experimental Animal Ethics committee of the Guangxi Medical University.

MSCs, which were obtained from bone marrow flushed out of the tibias and femurs of 4~6-week old C57 mice as described before [21], were cultured in α-minimal essential medium supplemented with 2 mM L-glutamine, 10% FBS and antibiotics (100 μg/ml streptomycin and 100 U/ml penicillin, both from Invitrogen). Non-adherent cells were removed after 72 h; while the rest adherent cells were maintained in media replenished every 3 days. Three passages later, about 3 × 10^6^ cells per mouse were obtained, and considered as purified MSCs and identified by adipocytes and osteoblasts differentiation as described in our previous studies [22–24]. Cells were used of their the 5th to 20th passage [25]. The MSCs were treated with vehicle or TNF-α and IFN-γ (20 ng/ml each, PeproTech) for 12 h before being collected for next step in vitro and in vivo experiments.

### Syngeneic prostate cancer mouse model

RM-1 cells were prepared either as mixing with MSCs treated with vehicle (1 × 10^6^ RM-1 cells and 2 × 10^5^ MSCs in 200 μl of phosphate buffer saline (PBS)) or mixing with MSCs treated with TNF-α and IFN-γ (1 × 10^6^ RM-1 cells and 2 × 10^5^ MSCs in 200 μl of PBS). RM-1 cells were administered subcutaneously in the armpit area of 6~8- week old C57 mice. Animals were sacrificed two weeks after tumor inoculation. Tumor volume was evaluated by the measuring the length and width of tumor mass.

### Conditioned medium

Being stimulated with TNF-α and IFN-γ (both 20 ng/ml) for 12 h, the culture medium of MSCs was replaced with fresh dulbecco’s modified eagle medium: nutrient mixture F-12 (DMEM F-12). And then being cultured for an additional 24 h, the conditioned medium was harvested and filtered through a 0.22 μm filtrator.

### Chorioallantoic membrane Angiogenic assay

This assay was performed follow a described method before [26]. Twenty chicken embryos which had hatched for 8 days, were randomly divided into two groups (10 for each group). After another 8 days hatching later, the air chambers of these chicken embryos were reopened. The number of each BV type was double blindly detected by three investigators following the criterion described before [27, 28].

### RNA extraction, reverse transcription, and real time PCR analyses

Total cellular mRNA was extract using Trizol Reagent (Invitrogen, Carlsbad, CA, USA). A 2 μg of total RNA was used to synthesize cDNA employing MMLV reverse transcriptase (Promega, WI, USA) and oligo dT-primers. PCR amplification was performed using 2 μL aliquots of cDNA. Real-time RT–PCR was carried out in triplicate using the SYBR PrimeScript RT–PCR Kit (Takara, Dalian, China). The primer sequences are listed in Table 1. Thermocycler conditions used as follow: 50 °C for 2 min and then 95 °C for 10 min, following by a two-step PCR program of 95 °C for 15 s and 60 °C for 60 s repeated for 40 cycles on an Mx4000 system (Stratagene, La Jolla, CA). The expression of β-actin was used as an internal control for normalization of the amount of RNA input. Normalized mRNA level was shown as fold change relative to the control sample.

**Table 1:** Oligonucleotide sequences used in real-time PCR and siRNA-mediated knockdown assay

Assay	Gene	Sequence (5′ → 3′)
Real-time PCR		
VEGF	F	GGA GAT CCT TCG AGG AGC ACT T
	R	GGC GAT TTA GCA GCA GAT ATA AGA A
PDGF	F	GCC GGT CCA GGT GAG AAA GAT TG
	R	GGG GCC GGC GGA TTC TCA
HIF-1α	F	CGG CGA AGC AAA GAG TCT GAA GT
	R	TCG CCG TCA TAT GTT AGC ACC AT
NRF2	F	CTCAGCATGATGGACTTGGA
	R	TCTATGTCTTGCCTCCAAAGG
β-Actin	F	CTC CAT CCT GGC CTC GCT GT
	R	GCT GTC ACC TTC ACC GTT CC
HIF-1α siRNA	(1)	CCC ATT CCT CAT CCG TCAA AT
	(2)	AGT CGA CAC AGC CTC GAT ATG
Control siRNA		UUC UCC GAA CGU GUC ACG UTT
NRF2 siRNA	(1)	GAAGGCACAATGGAATTCAAT
	(2)	GCCTTACTCTCCCAGTGAATA
Control siRNA		TTCTCCGAACGTGTCACGT

### Western blot analysis

After washing in PBS solution, total protein of cells was extracted using whole cell lysis buffer (Beyotime). Bio-Rad protein assay was applied to quantify the protein concentration. Immunoblotting was done as previously described [26]. The following antibodies were used: anti-HIF-1α, abti-NRF2 antibodies (1:500 polyclonal; Bethyl), anti-VEGF and anti-PDGF antibodies (1:1000 monoclonal; Abcam), and anti-rabbit peroxidase-conjugated secondary antibody (1:10,000; Sigma).

### Enzyme-linked Immunosorbent assay (ELISA)

ELISA assays were carried out using the commercial ELISA kit (VEGF, PDGF; R&D Systems). Assays were performed in technical duplicates and biological triplicates.

### Short interfering RNA (siRNA) and transient transfection

Oligoengine software was applied to design two siRNA sequences of HIF-1α and NRF2 (Table 1). Basic Local Alignment Search Tool (BLAST) was employed to confirm the specificity of these two sequences to their respective targets. Lipofectamine 2000 (Invitrogen) was used to perform transfections according to the manufacturer’s instructions. Cells (1–3 × 10^6^) in a confluence of 50–60% in 10 cm Petri dishes were transfected with siRNAs. These cells were harvested 48 h after transfection for RNA and protein analyses.

### Statistical analysis

Student’s *t*-test was applied to compare the mean values of two groups. Statistical analysis was done using GraphPad Prism 5 software. A *p*-value of less than 0.05 was considered to indicate a statistically significant difference.

## Results

### MSCs, stimulated with pro-inflammatory cytokines, increase the serum levels of pro-angiogenic factors and promote the growth of RM-1 prostate cancer cells in mice

**Fig. 1:**
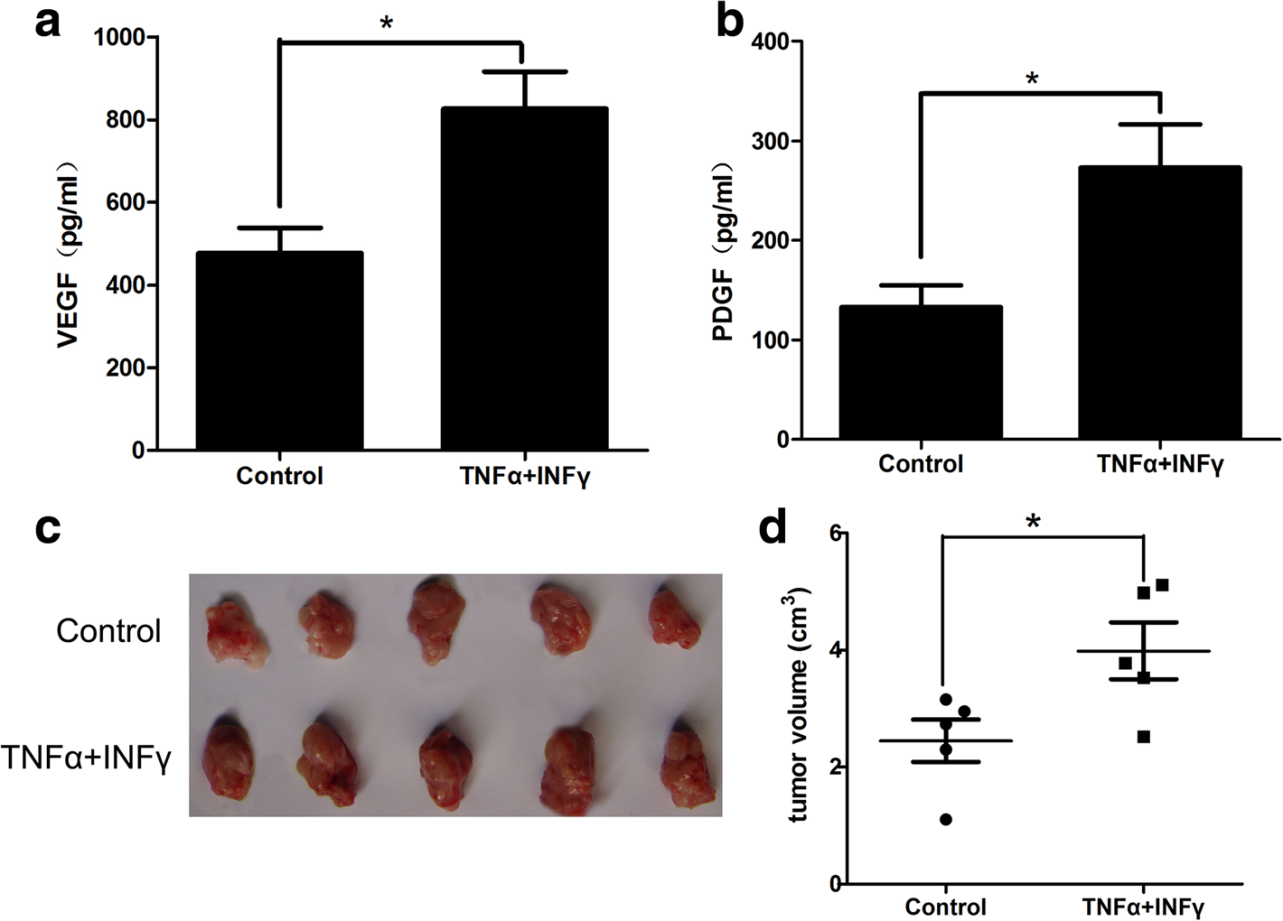
MSCs pretreated with inflammatory cytokines accelerate the growth of RM-1 prostate cancer cells in vivo by secreting of VEGF and PDGF. MSCs were pre-stimulated by inflammatory cytokines IFN-γ and TNF-α or vehicle for 12 h in advance, mixed with RM-1 cells (1 × 10^6^) and then subcutaneously administered in the armpit area of C57 mice. Total protein of VEGF and PDGF in the serum of mice was extracted at 3 days after implantation. All animals were sacrificed at 2 weeks. The volume of tumors was measured following removal of the tumors from the mice. **a** and **b** VEGF and PDGF protein levels were determined by ELISA analysis. **c** and **d** The tumor volume of mice subcutaneously implanted with RM-1 cells mixing with MSCs pretreated with IFN-γ and TNF-α or vehicle. ^*^ *p* < 0.05

We sought to elucidate the effects of MSCs on prostate tumor growth after they were stimulated by inflammatory cytokines. RM-1 cells mixing MSCs treated with TNF-α and IFN-γ for 12 h were co-injected C57 mice; RM-1 cells mixing with the MSCs treated with vehicle for 12 h was as the control group. ELISA was applied to measure the serum levels of PDGF and VEGF. Tumor growth volume was measured 14 days after tumor inoculation. As shown in Fig. 1a, the serum levels of VEGF were higher in mice receiving RM-1 cells and MSCs that were pretreated with TNF-α and IFN-γ than in the control group. Similar results were observed in the serum levels of PDGF (Fig. 1b). Consistently, RM-1 tumor volumes almost doubled in the group of mice that received RM-1 cells and cytokine-pretreated MSCs than in the control group (Fig. 1c and d). The results show that MSCs treated with TNF-α and IFN-γ promote the yielding of serum VEGF and PDGF and accelerate growth of RM-1 prostate tumors in vivo.

### MSCs pretreated with pro-inflammatory cytokines induce the expression of VEGF and PDGF and promote angiogenesis in chicken embryos

**Fig. 2:**
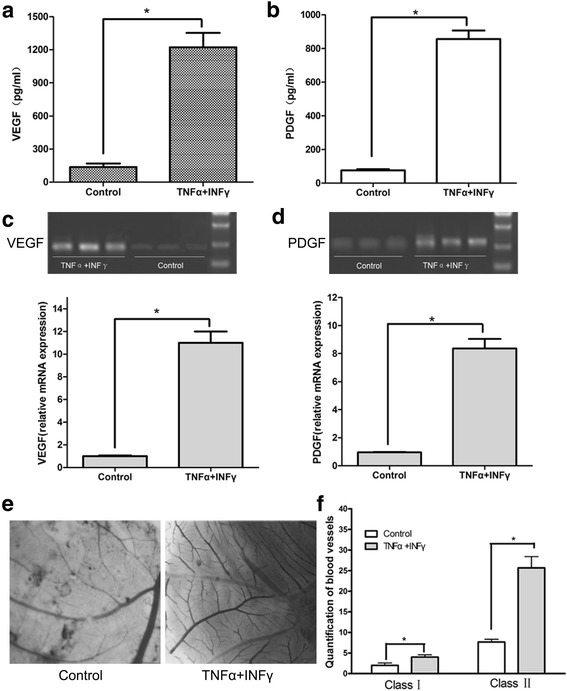
MSCs pretreated with inflammatory cytokines promote angiogenesis in chicken embryonic allantoides by producing of VEGF and PDGF. MSCs were pre-stimulated by inflammatory cytokines IFN-γ and TNF-α for 12 h. **a** and **b** ELISA analysis of the protein expression of VEGF and PDGF in the supernatant of MSCs. **c** and **d** Real-time PCR analysis of the mRNA expression of VEGF and PDGF in MSCs. BVs were broken down into two classes: bole vessels and branches on the bole vessels with vessel diameters not less than 1mm defined as class I BVs; branches on the bole vessels with vessel diameters less than 1mm were defined as class II BVs. **e** Embryonic allantois microvessels. **f** Quantification of chicken embryonic allantois microvessel formation. ^*^ *p* < 0.05

We next asked whether MSCs produced VEGF and PDGF in vitro and whether MSCs induce angiogenesis after pre-treatment with pro-inflammatory cytokines. We examined the protein levels of VEGF and PDGF in conditioned medium from MSCs treated with TNF-α and IFN-γ or vehicle control. As shown in Fig. 2a and b, TNF-α and IFN-γ pre-treatment raised the protein levels of VEGF and PDGF in the conditioned medium from MSCs. Consistent with increased secretion, the mRNA levels of VEGF and PDGF in MSCs were also increased (Fig. 2c and d). We next used a chick Chorioallantoic Membrane (CAM) assay to examine the angiogenic effect of conditioned medium from MSCs pre-treated with the pro-inflammatory cytokines. Conditioned medium from these MSCs were applied to the chick embryo as described in the Methods. The allantoides were fixed on cover slides and the BVs on the slides were counted after 8 days of hatching. As indicated in Fig. 2e and f, the MSC-conditioned medium from cells pre-treated with inflammatory cytokines exhibited stimulating effects in the formation of class I BVs. Moreover, greater stimulatory effect was observed in the formation of class II BVs. All these results indicate that MSCs increase the expression and secretion of pro-angiogenic factors and promote the formation of blood vessel under the stimulation with TNF-α and IFN-γ.

### NRF2-HIF-1α signaling controls the production of pro-angiogenic factors in MSCs pre-treated with pro-inflammatory cytokines

**Fig. 3:**
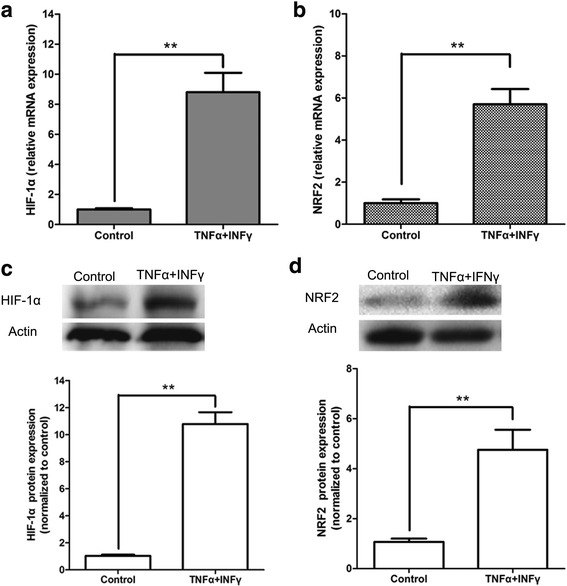
Expression of HIF-1α and NRF2 by MSCs pretreated with inflammatory factors. MSCs were pre-stimulated by inflammatory cytokines IFN-γ and TNF-α for 12 h. **a** Real-time PCR analysis of the expression of HIF-1α mRNA in MSCs. **b** Real-time PCR analysis of the expression of NRF2 mRNA in MSCs. **c** Western blot analysis of the protein expression of HIF-1α in MSCs. **d** Western blot analysis of the protein expression of NRF2 in MSCs.^**^ *p* < 0.01

So far, we showed that MSCs pre-treated with TNF-α and IFN-γ secreting two important pro-angiogenic factors, VEGF and PDGF. However, the underlying mechanism by which PDGF and VEGF levels increase in response to inflammatory cytokines is unknown. HIF-1α, an important transcription factor responsive to hypoxia, takes effect in the induction of many downstream angiogenesis-mediating genes, including PDGF and VEGF. NRF2 is an upstream regulatory gene of HIF-1α, and also believed to play an active role in tumor angiogenesis. Therefore, we examined the expression of HIF-1α and NRF2 (in mRNA and protein levels) in MSCs pretreated with TNF-α and IFN-γ. Compared to the control cells, MSCs pretreated with TNF-α and IFN-γ showed a substantial increase in HIF-1α and NRF2 mRNA (Fig. 3a and b) and protein (Fig. 3c and d).

**Fig. 4:**
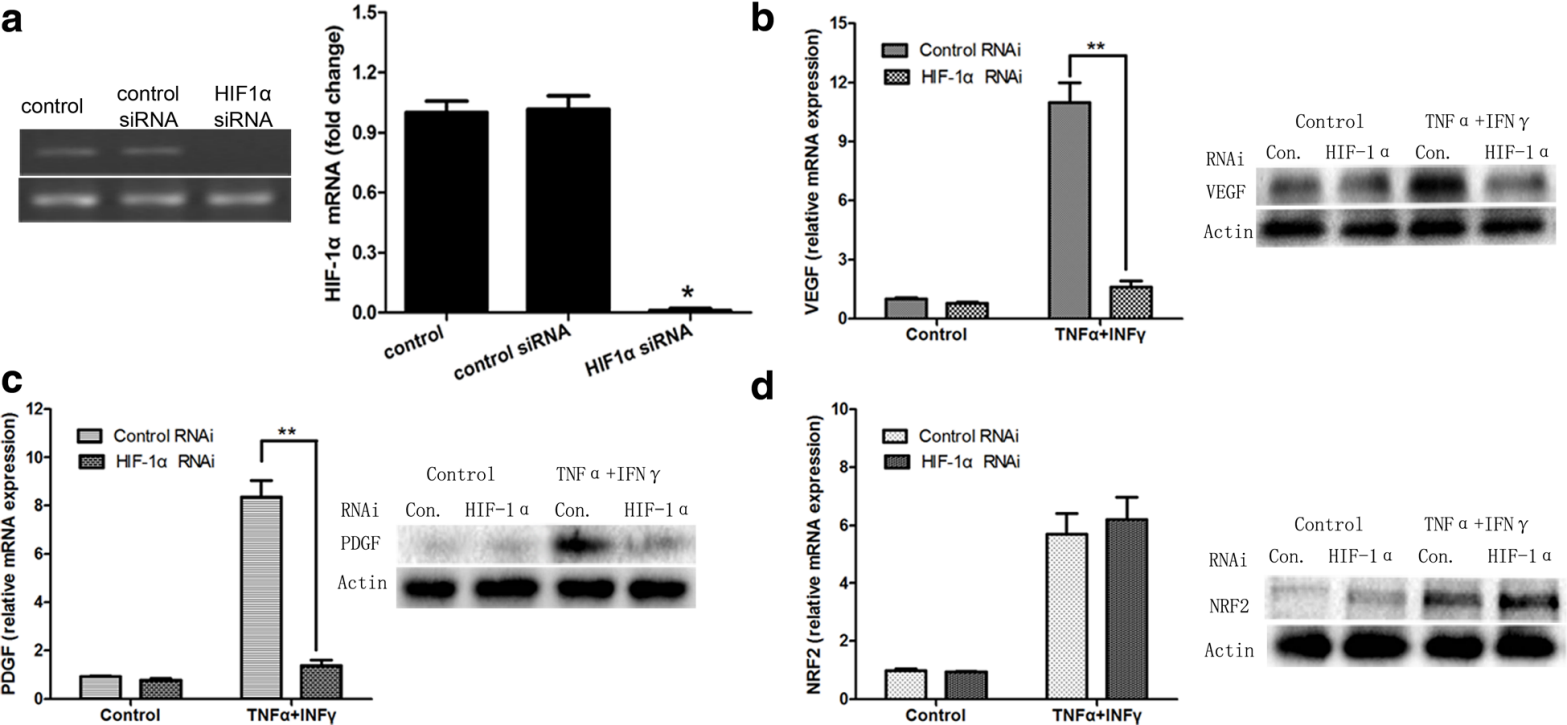
Regulation of the expression of VEGF, PDGF and NRF2 in MSCs by the HIF-1α signaling pathway. MSCs were pre-stimulated in advance by inflammatory cytokines IFN-γ and TNF-α for 12 h, then transfected with HIF-1α-siRNA sequence or the relative mock sequences. Total RNA was extracted at 24 h after infection. **a** Real-time PCR analysis of the mRNA expression of HIF-1α in MSCs that received no pretreatment and in MSCs treated with control siRNA or HIF-1α siRNA under inflammatory conditions (all groups were pretreated with IFN-γ and TNF-α). **b**, **c** and **d** Real-time PCR analysis of the mRNA and Western blot analysis of protein expression of VEGF, PDGF and NRF2 in MSCs that treated with control siRNA or HIF-1α siRNA under conditions pretreated with or without IFN-γ and TNF-α. ^*^ *p* < 0.05; ^**^ *p* < 0.01

We next asked whether NRF2-HIF-1α signaling is responsible for the induction of PDGF and VEGF in stimulated MSCs. This was achieved by measuring the expression of PDGF and VEGF after reducing the expression of HIF-1α or NRF2 in MSCs pretreated with TNF-α and IFN-γ. To knockdown the expression of HIF-1α, we transfected MSCs with siRNA against HIF-1α. As shown in Fig. 4a, HIF-1α siRNA significantly decreased the expression of HIF-1α mRNA in MSCs. The induction of PDGF and VEGF expression (mRNA and protein) was almost completely blocked in MSCs pretreated with TNF-α and IFN-γ when the expression of HIF-1α was inhibited by siRNA, while the expression of these genes in basal state (without cytokine treatment) did not obvious notable difference between the control and HIF-1α siRNA transfected cells (Fig. 4b and c). The levels of NRF2 mRNA and protein were insensitive to HIF-1α knockdown, consistent with NRF2 being the upstream regulator of HIF-1α (Fig. 4d).

**Fig. 5:**
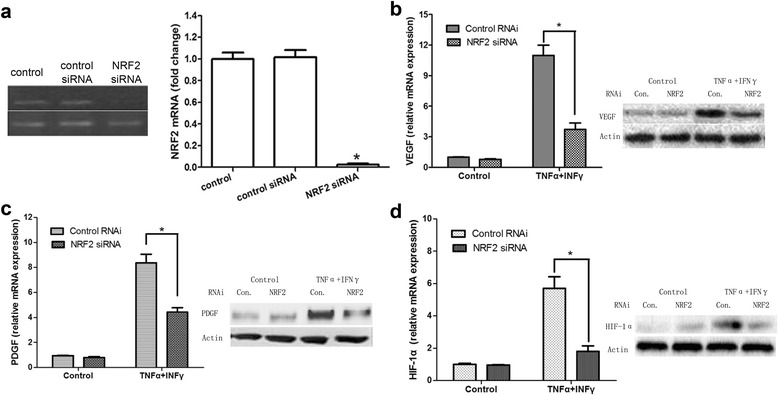
Regulation of the expression of VEGF,PDGF and HIF-1α in MSCs by the NRF2 signaling pathway. MSCs were pre-stimulated in advance by inflammatory cytokines IFN-γ and TNF-α for 12 h, then transfected with NRF2-siRNA sequence or the relative mock sequences. Total RNA was extracted at 24 h after infection. **a** Real-time PCR analysis of the mRNA expression of NRF2 in MSCs that received no pretreatment and in MSCs treated with control siRNA or NRF2 siRNA under inflammatory conditions (all groups were pretreated with IFN-γ and TNF-α). **b**, **c** and **d** Real-time PCR analysis of the mRNA and Western blot analysis of protein expression of VEGF, PDGF and HIF-1α in MSCs that treated with control siRNA or NRF2 siRNA under conditions pretreated with or without IFN-γ and TNF-α. ^*^ *p* < 0.05

Similar knockdown experiments were done to test the requirement of NRF2 on the induced expression of pro-angiogenic factors after pretreatment with TNF-α and IFN-γ (Fig. 5a). The mRNA and protein levels of PDGF and VEGF in control or NRF2 siRNA-transfected group did not exhibit an obvious change in control MSCs. However, when MSCs were stimulated previously by TNF-α and IFN-γ, NRF2 knockdown partially blocked the induction of mRNA and protein of PDGF and VEGF (Fig. 5b and c). Besides, NRF2 knockdown prevented the induction of expression of HIF-1α (mRNA and protein) (Fig. 5d). Collectively, these results showed that the NRF2- HIF-1α signaling pathway is required for pro-inflammatory cytokine-mediated induction of PDGF and VEGF in MSCs.

## Discussion

The prostate tissue is susceptible to infection and inflammation during a man’s lifetime. Chronic inflammation has been shown as not only an initiating event but also a development factor in prostate cancer. Substantial evidences show that the development of cancers from inflammation might be a process driven by inflammatory cells and a variety of mediators, such as cytokines, chemokines and enzymes, which altogether form an inflammatory microenvironment [29]. The pro-inflammatory chemokines, including Chemokine (C-C motif) ligand 5 (CCL5 /RANTES), stromal cell derived factor-1 (SDF-1/ CXCL12), and monocyte chemoattractant protein-1 (MCP-1/ CCL2), are often highly expressed in prostate cancer [30, 31]. TNF-α and IFN-γ are significant inflammatory cytokines affect tumor growth. Several studies have reported that TNF-α and IFN-γ synergize in modulating gene expression in some types of cells [32, 33], including MSCs [34]. The mechanism of this synergy in these two cytokines is not fully understood. It has been reported that C/EBPβ is likely to be a key transcription factor for regulating this synergy [35]. Our Additional file 1 shows the synergy of these two cytokines can result in up-regulation of C/EBPβ in MSCs. MSCs are often recruited to the tumor microenvironment due to local inflammation and may respond to pro-inflammatory cytokines to affect cancer progression. MSCs can promote tumor growth by increasing tumor vasculature through secreting the pro-angiogenic factors, including TGF-β, VEGF, PDGF, and basic fibroblast growth factor (bFGF). The pro-angiogenic and pro-cancerous effects of MSCs have been reported in a few types of solid cancers such as those from the colon [26], prostate and breast [36].

Consistent with the previous studies, we have observed that MSCs pre-stimulated by TNF-α and IFN-γ facilitated the growth of prostate cancer in a syngeneic mouse model, this tumor-promoting effect was accompanied by accumulation of VEGF and PDGF in the mouse serum. We showed that MSCs were the likely source of VEGF and PDGF since the mRNA expression and the secretion of these factors were dramatically increased in MSCs after treatment of TNF-α and IFN-γ. Conditioned medium of MSCs pretreated with TNF-α and IFN-γ enhanced angiogenesis in chicken embryonic allantoides. These results indicate that MSCs in the inflammatory microenviroment might produce pro-angiogenic factors, VEGF and PDGF, to enhance the angiogenesis of tumor and facilitate the growth of prostate cancer. We further showed that the increased expression and secretion of these pro-angiogenic factors required the NRF2-HIF-1α signaling, which was also elevated following treatment of pro-inflammatory cytokines in MSCs.

Angiogenesis is essential for tumor growth, specifically when tumor diameter is greater than 7 mm. As the tumor becomes enlarged, hypoxia is present in the inner side of the tumor. Besides, hypoxia is a common phenomenon at sites of inflammatory lesions and acts as a microenvironmental factor to enhance tumor angiogenesis by inducing HIF-1α and to accumulate reactive oxygen species (ROS) which is a potent inducer of NRF2 [37]. Therefore, NRF2-HIF-1α pathway likely takes effect during inflammation, by inducing the expression of a few pro-angiogenic target genes, including VEGF, PDGF, FGF, angiopoietins [38]. We had previously demonstrated that MSCs pre-treated by inflammatory cytokines such as TNF-α and IFN-γ in the tumor microenvironment express higher levels of VEGF via the HIF-1α signal pathway and promote colon cancer growth by enhancing tumor angiogenesis [26]. In a mouse xenograft model, Ji and co-workers reveal that knockdown of NRF2 inhibits the proliferation and growth of U251MG human glioma cells [39]. These results indicate that the NRF2-HIF-1α pathway might regulate angiogenesis by inducing the expression of PDGF and VEGF in prostate cancer.

In our study, MSCs pre-treated with TNF-α and IFN-γ induced much higher accumulation of HIF-1α and NRF2 protein and increased expression of mRNA of HIF-1α and NRF2. Besides, after knockdown of HIF-1α,the mRNA and protein levels of VEGF and PDGF were dramatically decreased in MSCs pretreated with TNF-α and IFN-γ,while the expression of NRF2 mRNA and protein did not change significantly. However, NRF2 blockade could down-regulate the mRNA and protein levels of VEGF, PDGF and HIF-1α. These findings indicate that the cross-talk between HIF-1α and NRF2 is responsible for inducing the expression of pro-angiogenic factors in MSCs pretreated with TNF-α and IFN-γ. Besides PDGF and VEGF, other factors might take effect in regulating tumor angiogenesis through NRF2-HIF-1α signaling pathway.

In the tumor microenvironment, MSCs also secrete a panel of growth, immunomodulatory, and signaling molecules, such as CCL5, CCL2, TGF-β, VEGF, IL-6, and IL-10, which may play role in angiogenesis. IL-8, also known as a pro-inflammatory chemokine, has been reported to play an active role in tumor angiogenesis in several tumors, including uterine cervical cancer, colon cancer, and pancreatic cancer [40–42]. IL-8 has been reported to be down-regulated by HIF-1α, while up-regulation of NRF2 could reverse the blocking effect of HIF-1α on IL-8 [43], suggesting that NRF2 may be involved in tumor angiogenesis through the IL-8 pathway. Our results provide new evidence that knockdown of NRF2 can suppress tumor angiogenesis by decreasing transcriptional activity of HIF-1α and inhibiting the expression of PDGF and VEGF gene. Moreover, the role of NRF2 cross-talk with HIF-1α in tumor angiogenesis was shown by previous studies. Ji et al. demonstrated that knockdown of NRF2 suppresses glioblastoma angiogenesis by inhibiting hypoxia-induced activation of HIF-1α [44]. Another study suggested that knockdown of NRF2 suppressed colon cancer growth in a mouse xenograft setting and was accompanied by a decrease in blood vessel formation and VEGF expression [17]. Collectively, these studies have shown that NRF2 inhibition can suppress tumor angiogenesis, possibly through inhibiting hypoxia-induced activation of HIF-1α signaling. More studies are required to reveal the precise mechanisms of NRF2 in tumor angiogenesis.

## Conclusions

In summary, our studies suggest that MSCs might promote prostate cancer growth in the inflammatory microenviroment through producing higher levels of PDGF and VEGF through the HIF-1α pathway and facilitating tumor angiogenesis. Knockdown of NRF2 decreases HIF-1α expression and inhibits the induction of pro-angiogenic factors following. Therefore, the pro-angiogenic effect of MSCs via the NRF2-HIF-1α pathway likely contributes to cancer growth in an inflammatory tumor environment. Our study suggests that NRF2 targeted therapy might be useful in treating prostate cancer.

## Additional file

Additional file 1: Involvement of C/EBPβ in IFN-γ and TNF-α induced HIF-1α expression. (A-B) MSCs were treated with IFN-γ, TNF-α, or both for 12 h and C/EBPβ levels were assessed by real-time PCR for mRNA and by Western blot analysis for protein. MSCs were transfected with C/EBPβ siRNA or a scrambled control siRNA. (C) After 12 h of IFN-γ and TNF-α stimulation, levels of expression of C/EBPβ were assessed by real-time PCR for mRNA and by Western blot analysis for protein. (D) C/EBPβ-siRNA-transfected MSCs were stimulated with IFN-γ, TNF-α, or both for 12 h and then HIF-1α-mRNA-assayed by real-time PCR.

## Abbreviations

bFGF: Basic fibroblast growth factor
BM-MSC: Bone marrow-derived mesenchymal stem cell
BVs: Blood vessels
CCL5 /RANTES: Chemokine (C-C motif) ligand 5
DMEM F-12: Dulbecco’s modified eagle medium: nutrient mixture F-12
ELISA: Enzyme-linked immunosorbent assay
FBS: Fetal bovine serum
HET-CAM: Hen egg test-chorioallantoic membrane
HIF-1α: Hypoxia-inducible factor-1 alpha
IFN-γ: Interferon gamma
IL-10: Interleukin-10
MCP-1/CCL2: Monocyte chemoattractant protein-1
MSC: Mesenchymal stem cells
NRF2: Nuclear factor-erythroid-2-related factor 2
PBS: Phosphate buffer saline
PCR: Polymerase chain reaction
PDGF: Platelet-derived growth factor
ROS: Reactive oxygen species
SDF-1/CXCL12: Stromal cell derived factor-1
siRNA: Short interfering RNA
TGF-β: Transforming growth factor-β
TNF-α: Tumor necrosis factor alpha
VEGF: Vascular endothelial growth factor

## References

[1] Jemal A, Siegel R, Xu J, Ward E (2010). Cancer statistics, 2010. CA Cancer J Clin.

[2] Drake CG (2010). Prostate cancer as a model for tumour immunotherapy. Nat Rev Immunol.

[3] Chiou SH, Sheu BC, Chang WC, Huang SC, Hong-Nerng H (2005). Current concepts of tumor-infiltrating lymphocytes in human malignancies. J Reprod Immunol.

[4] De Nunzio C, Kramer G, Marberger M, Montironi R, Nelson W, Schroder F, Sciarra A, Tubaro A (2011). The controversial relationship between benign prostatic hyperplasia and prostate cancer: the role of inflammation. Eur Urol.

[5] Lorusso G, Ruegg C (2008). The tumor microenvironment and its contribution to tumor evolution toward metastasis. Histochem Cell Biol.

[6] Orimo A, Gupta PB, Sgroi DC, Arenzana-Seisdedos F, Delaunay T, Naeem R, Carey VJ, Richardson AL, Weinberg RA (2005). Stromal fibroblasts present in invasive human breast carcinomas promote tumor growth and angiogenesis through elevated SDF-1/CXCL12 secretion. Cell.

[7] Kim S, Takahashi H, Lin WW, Descargues P, Grivennikov S, Kim Y, Luo JL, Karin M (2009). Carcinoma-produced factors activate myeloid cells through TLR2 to stimulate metastasis. Nature.

[8] Whiteside TL (2008). The tumor microenvironment and its role in promoting tumor growth. Oncogene.

[9] Szlosarek P, Charles KA, Balkwill FR (2006). Tumour necrosis factor-alpha as a tumour promoter. Eur J Cancer.

[10] Yang CH, Murti A, Pfeffer LM (2005). Interferon induces NF-kappa B-inducing kinase/tumor necrosis factor receptor-associated factor-dependent NF-kappa B activation to promote cell survival. J Biol Chem.

[11] Calon A, Espinet E, Palomo-Ponce S, Tauriello DV, Iglesias M, Cespedes MV, Sevillano M, Nadal C, Jung P, Zhang XH (2012). Dependency of colorectal cancer on a TGF-beta-driven program in stromal cells for metastasis initiation. Cancer Cell.

[12] Cuiffo BG, Karnoub AE (2012). Mesenchymal stem cells in tumor development: emerging roles and concepts. Cell Adhes Migr.

[13] Kong T, Eltzschig HK, Karhausen J, Colgan SP, Shelley CS (2004). Leukocyte adhesion during hypoxia is mediated by HIF-1-dependent induction of beta2 integrin gene expression. Proc Natl Acad Sci United States of America.

[14] Menegon S, Columbano A, Giordano S. The dual roles of NRF2 in cancer. Trends Mol Med. 2016; 10.1016/j.molmed.2016.05.002 27263465

[15] Gan N, Sun X, Song L (2010). Activation of Nrf2 by microcystin-LR provides advantages for liver cancer cell growth. Chem Res Toxicol.

[16] Shibata T, Kokubu A, Saito S, Narisawa-Saito M, Sasaki H, Aoyagi K, Yoshimatsu Y, Tachimori Y, Kushima R, Kiyono T (2011). NRF2 mutation confers malignant potential and resistance to chemoradiation therapy in advanced esophageal squamous cancer. Neoplasia.

[17] Kim TH, Hur EG, Kang SJ, Kim JA, Thapa D, Lee YM, Ku SK, Jung Y, Kwak MK (2011). NRF2 blockade suppresses colon tumor angiogenesis by inhibiting hypoxia-induced activation of HIF-1alpha. Cancer Res.

[18] Homma S, Ishii Y, Morishima Y, Yamadori T, Matsuno Y, Haraguchi N, Kikuchi N, Satoh H, Sakamoto T, Hizawa N (2009). Nrf2 enhances cell proliferation and resistance to anticancer drugs in human lung cancer. Clin Can Res.

[19] Nelson WG, De Marzo AM, Isaacs WB (2003). Prostate cancer. N Engl J Med.

[20] De Marzo AM, Platz EA, Sutcliffe S, Xu J, Gronberg H, Drake CG, Nakai Y, Isaacs WB, Nelson WG (2007). Inflammation in prostate carcinogenesis. Nat Rev Cancer.

[21] Zhu H, Guo ZK, Jiang XX, Li H, Wang XY, Yao HY, Zhang Y, Mao N (2010). A protocol for isolation and culture of mesenchymal stem cells from mouse compact bone. Nat Protoc.

[22] Cheng J, Ye H, Liu Z, Xu C, Zhang Z, Liu Y, Sun Y (2013). Platelet-derived growth factor-BB accelerates prostate cancer growth by promoting the proliferation of mesenchymal stem cells. J Cell Biochem.

[23] Yang X, Han ZP, Zhang SS, Zhu PX, Hao C, Fan TT, Yang Y, Li L, Shi YF, Wei LX (2014). Chronic restraint stress decreases the repair potential from mesenchymal stem cells on liver injury by inhibiting TGF-beta1 generation. Cell Death Dis.

[24] Hou J, Han ZP, Jing YY, Yang X, Zhang SS, Sun K, Hao C, Meng Y, Yu FH, Liu XQ (2013). Autophagy prevents irradiation injury and maintains stemness through decreasing ROS generation in mesenchymal stem cells. Cell Death Dis.

[25] Alhadlaq A, Mao JJ (2004). Mesenchymal stem cells: isolation and therapeutics. Stem Cells Dev.

[26] Liu Y, Han ZP, Zhang SS, Jing YY, Bu XX, Wang CY, Sun K, Jiang GC, Zhao X, Li R (2011). Effects of inflammatory factors on mesenchymal stem cells and their role in the promotion of tumor angiogenesis in colon cancer. J Biol Chem.

[27] DeFouw DO, Rizzo VJ, Steinfeld R, Feinberg RN (1989). Mapping of the microcirculation in the chick chorioallantoic membrane during normal angiogenesis. Microvasc Res.

[28] Rizzo V, DeFouw DO (1996). Mast cell activation accelerates the normal rate of angiogenesis in the chick chorioallantoic membrane. Microvasc Res.

[29] Coussens LM, Werb Z (2002). Inflammation and cancer. Nature.

[30] Vaday GG, Peehl DM, Kadam PA, Lawrence DM (2006). Expression of CCL5 (RANTES) and CCR5 in prostate cancer. Prostate.

[31] Fujita K, Ewing CM, Getzenberg RH, Parsons JK, Isaacs WB, Pavlovich CP (2010). Monocyte chemotactic protein-1 (MCP-1/CCL2) is associated with prostatic growth dysregulation and benign prostatic hyperplasia. Prostate.

[32] Sekine N, Ishikawa T, Okazaki T, Hayashi M, Wollheim CB, Fujita T (2000). Synergistic activation of NF-kappab and inducible isoform of nitric oxide synthase induction by interferon-gamma and tumor necrosis factor-alpha in INS-1 cells. J Cell Physiol.

[33] Lee AH, Hong JH, Seo YS (2000). Tumour necrosis factor-alpha and interferon-gamma synergistically activate the RANTES promoter through nuclear factor kappaB and interferon regulatory factor 1 (IRF-1) transcription factors. Biochem J.

[34] English K, Barry FP, Field-Corbett CP, Mahon BP (2007). IFN-gamma and TNF-alpha differentially regulate immunomodulation by murine mesenchymal stem cells. Immunol Lett.

[35] Xu G, Zhang Y, Zhang L, Roberts AI, Shi Y (2009). C/EBPbeta mediates synergistic upregulation of gene expression by interferon-gamma and tumor necrosis factor-alpha in bone marrow-derived mesenchymal stem cells. Stem Cells.

[36] Zhang T, Lee YW, Rui YF, Cheng TY, Jiang XH, Li G (2013). Bone marrow-derived mesenchymal stem cells promote growth and angiogenesis of breast and prostate tumors. Stem Cell Res Ther.

[37] Guzy RD, Hoyos B, Robin E, Chen H, Liu L, Mansfield KD, Simon MC, Hammerling U, Schumacker PT (2005). Mitochondrial complex III is required for hypoxia-induced ROS production and cellular oxygen sensing. Cell Metab.

[38] Du R, Lu KV, Petritsch C, Liu P, Ganss R, Passegue E, Song H, Vandenberg S, Johnson RS, Werb Z (2008). HIF1alpha induces the recruitment of bone marrow-derived vascular modulatory cells to regulate tumor angiogenesis and invasion. Cancer Cell.

[39] Ji XJ, Chen SH, Zhu L, Pan H, Zhou Y, Li W, You WC, Gao CC, Zhu JH, Jiang K (2013). Knockdown of NF-E2-related factor 2 inhibits the proliferation and growth of U251MG human glioma cells in a mouse xenograft model. Oncol Rep.

[40] Li A, Varney ML, Singh RK (2001). Expression of interleukin 8 and its receptors in human colon carcinoma cells with different metastatic potentials. Clin Cancer Res.

[41] Matsuo Y, Ochi N, Sawai H, Yasuda A, Takahashi H, Funahashi H, Takeyama H, Tong Z, Guha S (2009). CXCL8/IL-8 and CXCL12/SDF-1alpha co-operatively promote invasiveness and angiogenesis in pancreatic cancer. Int J Cancer.

[42] Chen RJ, Chen SU, Chou CH, Lin MC (2012). Lysophosphatidic acid receptor 2/3-mediated IL-8-dependent angiogenesis in cervical cancer cells. Int J Cancer.

[43] Loboda A, Stachurska A, Florczyk U, Rudnicka D, Jazwa A, Wegrzyn J, Kozakowska M, Stalinska K, Poellinger L, Levonen AL (2009). HIF-1 induction attenuates Nrf2-dependent IL-8 expression in human endothelial cells. Antioxid Redox Signal.

[44] Ji X, Wang H, Zhu J, Zhu L, Pan H, Li W, Zhou Y, Cong Z, Yan F, Chen S (2014). Knockdown of Nrf2 suppresses glioblastoma angiogenesis by inhibiting hypoxia-induced activation of HIF-1alpha. Int J Cancer.

