# Supplementary figures and images for: Bone marrow-derived mesenchymal stem cells induced by inflammatory cytokines produce angiogenetic factors and promote prostate cancer growth
Source: BMC Cancer. 2017 Dec 21;17:878. doi: 10.1186/s12885-017-3879-z (PMC5740893; doi:10.1186/s12885-017-3879-z)

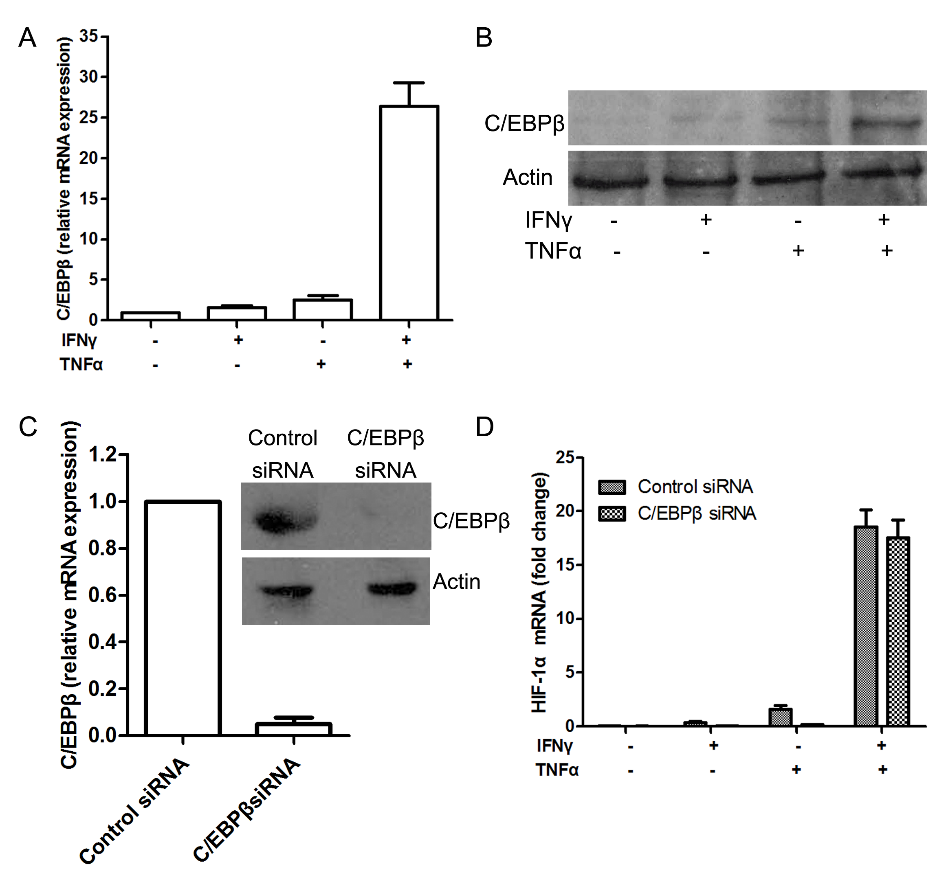

Supplement: Additional file 1: — Involvement of C/EBPβ in IFN-γ and TNF-α induced HIF-1α expression. (A-B) MSCs were treated with IFN-γ, TNF-α, or both for 12 h and C/EBPβ levels were assessed by real-time PCR for mRNA and by Western blot analysis for protein. MSCs were transfected with C/EBPβ siRNA or a scrambled control siRNA. (C) After 12 h of IFN-γ and TNF-α stimulation, levels of expression of C/EBPβ were assessed by real-time PCR for mRNA and by Western blot analysis for protein. (D) C/EBPβ-siRNA-transfected MSCs were stimulated with IFN-γ, TNF-α, or both for 12 h and then HIF-1α-mRNA-assayed by real-time PCR. [file 12885_2017_3879_MOESM1_ESM.docx]
